# Oral and Intravenous Fosfomycin for the Treatment of Complicated Urinary Tract Infections

**DOI:** 10.1155/2020/8513405

**Published:** 2020-03-28

**Authors:** George G. Zhanel, Michael A. Zhanel, James A. Karlowsky

**Affiliations:** Department of Medical Microbiology and Infectious Diseases, Max Rady College of Medicine, University of Manitoba, Winnipeg, Manitoba, Canada

## Abstract

Oral fosfomycin is approved in Canada for the treatment of acute uncomplicated cystitis. Several studies have reported “off label” use of oral fosfomycin in the treatment of patients with complicated lower urinary tract infection (cLUTI). This review summarizes the available literature describing the use of oral fosfomycin in the treatment of patients with cLUTI. Collectively, these studies support the use of a regimen of 3 grams of oral fosfomycin administered once every 48 or 72 hours for a total of 3 doses for patients who have previously failed treatment with another agent, are infected with a multidrug-resistant (MDR) pathogen, or cannot tolerate first-line treatment due to intolerance or adverse effects. Additionally, a Phase 2/3 clinical trial, known as the ZEUS study, assessed the efficacy and safety of intravenous (IV) fosfomycin versus piperacillin-tazobactam in the treatment of patients with complicated upper urinary tract infection (cUUTI) or acute pyelonephritis (AP) including in patients with concomitant bacteremia. IV fosfomycin was reported to be noninferior to piperacillin-tazobactam in treating patients with cUUTI and AP; however, when outcomes were independently evaluated according to baseline diagnosis (i.e., cUUTI versus AP), IV fosfomycin was superior to piperacillin-tazobactam in the treatment of patients with cUUTI and demonstrated superior microbiological eradication rates, across all resistant phenotypes including extended-spectrum *β*-lactamase- (ESBL-) producing *Escherichia coli* and *Klebsiella* spp. and carbapenem-resistant (CRE), aminoglycoside-resistant, and MDR Gram-negative bacilli (primarily *Enterobacterales*). Based on the ZEUS study, IV fosfomycin dosed at 6 grams every 8 hours for 7 days (14 days in patients with concurrent bacteremia) appears to be a safe and effective therapeutic option in treating patients with upper urinary tract infections, particularly those with cUUTI caused by antimicrobial-resistant *Enterobacterales*.

## 1. Introduction

Fosfomycin demonstrates potent *in vitro* activity against *Escherichia coli* and other common uropathogens, including extended-spectrum *β*-lactamase- (ESBL-) producing, AmpC-producing, carbapenem-resistant, and multidrug-resistant (MDR) isolates of *Enterobacterales* [1]. Antimicrobial resistance to fosfomycin is rare in *E. coli* (<1%), while resistance to fluoroquinolones and trimethoprim-sulfamethoxazole is frequent (>20%) and continues to increase [2]. In 2013, oral fosfomycin was approved in Canada for the treatment of acute uncomplicated cystitis. For this indication, fosfomycin is administered as a single 3 gram oral dose, regardless of the patient's renal or hepatic function. Clinically, fosfomycin has demonstrated efficacy and safety in the treatment of acute uncomplicated cystitis and is well-tolerated in pregnancy and in the elderly [1]. However, oral fosfomycin is a frequently used “off indication” or “off label” to treat patients with complicated lower urinary tract infection (cLUTI). In addition, intravenous (IV) fosfomycin has recently been studied in a Phase 2/3 clinical trial for the treatment of patients with complicated upper urinary tract infection (cUUTI) and acute pyelonephritis (AP) [3].

The purpose of this paper is to provide a review of the available literature regarding the clinical use of oral fosfomycin in the treatment of cLUTIs and to make recommendations about when and how this agent may be used. As well, we will review the available literature regarding the clinical use of IV fosfomycin in the treatment of cUUTI and AP to make recommendations about when and how this agent may be used. We reviewed the available literature on the use of oral fosfomycin in the treatment of cLUTI from 1987 to June 2019, inclusive, and the recent IV fosfomycin literature for its use in the treatment of cUUTI and AP.

## 2. Definition of Complicated Lower Urinary Tract Infection (cLUTI) and Complicated Upper Urinary Tract Infection (cUUTI)

For the purpose of this review, we have defined a patient with cLUTI as a patient with typical signs and symptoms of LUTI and one or more of the following complicating factor(s): (i) infection due to a MDR bacterial pathogen, (ii) urinary instrumentation (e.g., urinary catheterization), (iii) history of recurrent UTI, (iv) male gender, (v) renal transplant, (vi) renal impairment including acute or chronic kidney disease, (vii) functional/anatomical abnormality causing renal obstruction (e.g., nephrolithiasis), (viii) urinary retention due to neurogenic bladder, hemiparesis or quadriparesis, or prostatic hypertrophy, (ix) malignancy involving the urinary tract or another site, (x) pregnancy, (xi) chronic systemic disease (e.g., diabetes mellitus), (xii) immunosuppression/corticosteroid use, (xiii) hospitalization, and (xiv) recent urological intervention.

We defined cUUTI by the criteria used in the ZEUS Phase 2/3 clinical trial [3]. The criteria applied to hospitalized patients with typical signs and symptoms of UUTI/AP and one or more associated risk factors including: (i) use of intermittent bladder catheterization or presence of an indwelling bladder catheter, (ii) current and known functional or anatomical abnormality of the urogenital tract (including anatomic malformations or neurogenic bladder or patients with a post-void residual urine volume of ≥100 mL), (iii) complete or partial obstructive uropathy (e.g., nephrolithiasis, tumor, fibrosis, and urethral stricture) that was expected to be medically or surgically treated during study drug therapy, and (iv) azotemia (defined as blood urea nitrogen (BUN) >20 mg/dL, blood urea >42.8 mg/dL, or serum creatinine >1.4 mg/dL) due to known prior intrinsic renal disease or chronic urinary retention in men (e.g., men previously diagnosed with benign prostatic hypertrophy) [3].

## 3. Oral Fosfomycin for the Treatment of Complicated Lower Urinary Tract Infection (cLUTI)

Several studies have been published citing the offlabel use of oral fosfomycin in the treatment of cLUTI (Table 1). Moroni et al. published the first of these studies in 1987 as a subset analysis in a multicentre, open, noncontrolled trial that included a total of 365 patients with clinical signs and symptoms of LUTI and significant bacteriuria by urine culture [4]. Of the 365 patients, 49 patients (20 male and 29 female) were categorized as having a cLUTI infection, although Moroni et al. did not specify the definition of cLUTI in their paper. Twenty-one of the 49 patients (42.9%) with cLUTI were treated with a single 3-gram dose of oral fosfomycin, while the remaining 28 patients were treated with a multidose regimen consisting of 3 grams of fosfomycin once daily for 2-3 days or in a few cases, 3 grams once daily for 11 days. Clinical cure was reported in 57.1% (12/21) of patients treated with a single-dose regimen of fosfomycin compared to 82.1% (23/28) of patients treated with multiple doses. Clinical cure was defined as resolution of symptoms and a negative urine cultures at the 3 weeks follow-up. Based on these data, Moroni et al. suggested treatment courses of at least 3 days for cLUTIs should be used. Fosfomycin was reported to be well-tolerated in both single and multidose treatment courses. Despite minor limitations in the study design and an ambiguous definition of cLUTI, this study suggests that oral fosfomycin may be a potential therapeutic option in treatment of cLUTIs.

In 2007, Pullukcu et al. published a retrospective study evaluating 52 patients with LUTIs caused by ESBL-producing *E. coli* treated with multiple 3-gram oral doses of fosfomycin [5] (Table 1). Inclusion criteria in this study were patients >18 years of age with dysuria, frequency, or urgency in passing urine, >20 leukocytes/mL by urine microscopy, culture proven ESBL-producing *E. coli* in urine (>10^5^ CFU/mL), and no leukocytosis or fever. All patients were treated with 3 grams of oral fosfomycin every other day for a total of 3 doses. The study population consisted of 25 males and 27 females with an average age of 55 ± 18.3 years and an age range of 19–85 years. Of the 52 patients, 36 had an additional complicating factor including 7 patients with an indwelling urinary catheter, 6 patients that had undergone a recent urological intervention, 5 patients with diabetes mellitus, 5 renal transplant patients, 4 patients with malignancy involving the urinary tract, 4 patients with another malignancy, 3 patients with nephrolithiasis, and 2 patients having hemiparesis or quadraparesis. Clinical cure was defined as resolution of symptoms and microbiological cure was defined as a sterile urine culture at 7–9 days follow-up. Overall, the clinical and microbiological cure rates were reported as 94.3% (49/52) and 78.5% (41/52), respectively [5]. Relapse and reinfection was assessed by a supplementary urine culture obtained 28 days after completion of therapy. Relapse was defined as isolation of ESBL-producing *E. coli* while reinfection was defined as isolation of any pathogen [5]. Of the 41 patients deemed to have microbiological cure, 68.2% (28/41) had a urine culture taken at 28 days after therapy. Relapse and reinfection rates were 0% (0/28) and 10.7% (3/28), respectively. The authors concluded that fosfomycin may be a suitable, effective, and cheap alternative in the treatment of ESBL-producing *E. coli*-related LUTIs.

Senol et al. published an observational prospective study in 2010 comparing the efficacy of oral fosomycin against IV carbapenems in the treatment of cLUTI caused by ESBL-producing *E. coli* [6]. Inclusion criteria were patients >18 years of age with dysuria or problems with frequency or urgency in passing urine, >20 leukocytes/mL by urine microscopy, culture proven ESBL-producing *E. coli* in the urine (>10^5^ CFU/mL), and no leukocytosis or fever [6]. Patients in the study were considered to have a cLUTI in cases where at least one or more of the following complicating factors were present: an indwelling urinary catheter, diabetes mellitus, neurogenic bladder, obstruction due to nephrolithiasis or fibrosis, urinary retention due to benign prostatic hypertrophy, bladder cancer, or other urologic abnormality. Patients were treated with either 3 grams of oral fosfomycin every other day for a total of 3 doses, or IV meropenem 1 gram thrice daily, or IV imipenem/cilastatin 500 mg 4 times daily for 14 days [6]. The study population consisted of a total of 47 patients (27 treated with fosfomycin and 20 treated with a carbapenem). The fosfomycin group consisted of 13 males and 14 females with an average age of 57.5 ± 15.3 years. Patients in the fosfomycin group had an average of 1.7 ± 0.5 complicating factors with 19 patients having >1 complicating factor. The carbapenem group was composed of patients with similar gender composition, age, and complicating factors. Clinical cure was defined as resolution of symptoms, while microbiological cure was defined as a sterile urine culture at 7–9 days follow-up [6]. Overall, the clinical and microbiological cure rates in the fosfomycin group were 77.8% (21/27) and 59.3% (16/27), respectively. Clinical and microbiological cure rates were similar in the carbapenem group. Relapse and reinfection were assessed by a urine culture obtained 28–30 days after completion of therapy. Relapse was defined as isolation of ESBL-producing *E. coli*, while reinfection was defined as isolation of any pathogen. Of the 16 patients deemed to have microbiological cure in the fosfomycin group, all had a supplementary urine culture taken at 28–30 days after treatment. Relapse and reinfection rates were both 6.3% (1/16). The authors concluded that oral fosfomycin was a suitable, effective, and cheap alternative in the treatment of ESBL-producing *E. coli*-associated cLUTIs.

Neuner et al. published a retrospective chart review in 2012 assessing clinical outcomes of hospitalized patients with LUTIs caused by MDR urinary pathogens treated with oral fosfomycin [7]. All 41 patients (19 males, 22 females; mean age of 62 ± 15 years) included in the review had a positive urine culture that grew an MDR uropathogen and received at least one 3-gram dose of fosfomycin in hospital. An abnormal urinalysis or symptoms of LUTI, in addition to a positive urine culture, were required to meet criteria for LUTI. A total of 44 urinary pathogens were isolated from 41 patients including 13 carbapenem-resistant *Klebsiella pneumoniae*, 8 *Pseudomonas aeruginosa*, 7 vancomycin-resistant *Enterococcus faecium* (VRE), 7 ESBL-producing *E. coli* and *K. pneumo*niae, and 9 others (5 *E. coli*, 1 *Acinetobacter baumannii*, 1 *Enterobacter cloacae*, 1 *Enterococcus faecalis*, and 1 *Proteus mirabilis*). Patients in this study had significant complicating factors/comorbidities (average of 4.4 complicating factors/comorbidities per patient). Eighty percent of patients had ≥1 complicating factor in addition to infection by a MDR urinary pathogen. The most common complicating factors included urinary catheterization (63.4% of patients), a history of recurrent LUTI (24.4% of patients), and urological surgery within the last 6 months (14.6% of patients) [7]. Other complicating factors included a suprapubic catheter, ureteral stent, percutaneous nephrostomy tube, umbilical stoma, and neurogenic bladder. Patients were treated with an average of 2.9 ± 1.8 3 gram oral doses of fosfomycin with varying regimens depending on clinical circumstances; 11/41 patients (26.8%) were treated with fosfomycin in combination with other antimicrobials. Microbiological cure, defined as a negative urine culture after completion of therapy and/or absence of relapse or reinfection, was reported in 58.5% (24/41) of patients. The authors noted that there were significantly more solid organ transplant recipients in the microbiological failure group (59% versus 21%; *p*=0.02). Relapse and reinfection occurred in 24.4% (10/41) and 17.1% (7/41) of patients, respectively. Relapse was defined as isolation of the same pathogen, while reinfection was defined as isolation of any pathogen at a 30-day follow-up visit. The authors concluded that fosfomycin showed good *in vitro* activity against MDR urinary pathogens; however, they cautioned use of oral fosfomycin in transplant recipients, especially in kidney transplant recipients.

In 2013, Qiao et al. published a prospective, uncontrolled, open label study assessing the efficacy of oral fosfomycin in the treatment of UTIs including ‘recurrent LUTIs' and cLUTIs [8]. The study evaluated a total of 361 patients (114 males, 247 females; mean age 49.63 ± 16.64 years), of which 146 patients were categorized as having either recurrent LUTI or cLUTI in their per-protocol set. We pooled the data for recurrent LUTI and cLUTI to comply with our definition of cLUTI. All patients were treated with 3 grams oral fosfomycin every other day for a total of 3 doses. Overall, the clinical cure rate for patients with recurrent LUTI or cLUTIs was 70.5% (77.2% for recurrent LUTI and 62.7% for cLUTI). The overall microbiological cure rate was 74.6% (75.0% for recurrent LUTIs and 74.2% for cLUTIs). Relapse or reinfection occurred in 14.9% of cases. The authors concluded that oral fosfomycin demonstrated clinical and microbiological efficacy in the treatment of recurrent and cLUTIs.

In 2016, Matthews et al., published a retrospective study evaluating a group of patients with complex, recurrent, resistant, or persistent LUTIs treated with oral fosfomycin [9]. Seventy-five patients (18 males, 57 females; median age 73 years) were included in the study, of which 69.3% (52/75) had a complicating factor/comorbidity and 49.3% (37/75) were infected with MDR pathogens. Complicating factors/comorbidities included: 25 patients with genitourinary tract pathology (stones, cancer of prostate/bladder/kidneys, urethral disease, and self-catheterization), 12 renal transplant patients, 12 patients with systemic disease (non-renal tract malignancy, steroids, diabetes, cardiovascular disease, and gastrointestinal tract disease), and 3 pregnant patients. The majority of patients, 69.3% (52/75), had infections due to *E. coli*, of which 58.5% (31/52) were ESBL-producing *E. coli*; 66.7% (6/9) of *K. pneumoniae* isolates were also ESBL-producers. 70.6% (53/75) of patients were treated with a single 3-gram dose of oral fosfomycin, 10.6% (8/75) received two 3-gram doses, and 18.7% (14/75) received ≥3 oral 3 gram doses of fosfomycin (Table 1). Several patients received numerous 3-gram doses of fosfomycin over an extended period of time, with no serious side effects. Success of treatment was measured using two different definitions, “functional cure” and “microbiological cure.” Functional cure included patients with a negative urine culture or those in which a follow-up urine sample was unavailable (presumably due to resolution of symptoms, i.e., clinical cure). Follow-up occurred at a median duration of 13 days after the initial dose of fosfomycin. Microbiological cure was defined in cases where a follow-up urine culture was obtained and the culture was negative. Functional cure was reported in 68.9% (42/61) patients and microbiological cure in 52.5% (21/40) of patients. Reinfection was defined as isolation of a different urinary pathogen at follow-up compared to pretreatment cultures. 10.7% (8/75) of patients met the definition for reinfection. Of interest, patients with *Klebsiella* spp. infections were more likely to fail treatment than those with infections due to *E. coli*. The authors concluded that fosfomycin is safe and effective in the treatment of LUTIs in patients with complex comorbidities and overextended treatment durations.

In 2016, Veve et al. published a retrospective cohort study analyzing the efficacy of oral fosfomycin versus IV ertapenem in the treatment of ESBL-associated LUTIs [10]. Inclusion criteria in this study were patients ≥18 years of age, receiving outpatient treatment with fosfomycin or ertapenem for a symptomatic UTI, a positive urine culture for *E. coli*, *Klebsiella* spp. or another species of *Enterobacterales* shown to resistant to penicillins, monobactams, and oxyimino-cephalosporins, and confirmed to be ESBL-producing using a phenotypic method. A total of 178 patients were included in the study, 89 of which were treated with oral fosfomycin and 89 with IV ertapenem. The fosfomycin group consisted of 23 males and 66 females, with a mean age of 69.3 ± 17.9 years. The ertapenem group consisted of 51 males and 38 females, with a mean age of 69.2 ± 17.7 years. 83.7% (149/178) of patients had infection due to ESBL-producing *E. coli*, 14.6% (26/178) had infections due to ESBL-producing *Klebsiella* spp., and 3 patients had infections due to other ESBL-producing *Enterobacterales*. The most common complicating factors/comorbidities in patients treated in the fosfomycin group were acute or chronic kidney disease which was present in 41.6% (37/89) of patients, and urinary catheterization which was present in 38.2% (34/89) of patients. Other complicating factors/comorbidities included the presence of chronic systemic disease such as cardiovascular disease, diabetes, pulmonary disease, previous nephrectomy or history of urological procedure, paraplegia or quadriplegia, renal calculi or nephrolithiasis, immunosuppression or steroid use, and history of renal transplant. Fosfomycin oral dosing regimens included a 3-gram single dose in 12/89 (13.5%) patients, 3 grams every 48 hours for an average duration of 6 days (i.e., ∼3 doses total) in 20/89 (22.5%) patients, 3 grams every 72 hours for an average duration of 9 days (i.e., ∼3 doses total) in 55/89 (61.8%) patients, and a 3-gram daily dose for an average duration of 4.5 days in 2/89 (2.2%) patients. Ertapenem was predominantly administered as 1 gram IV every 24 hours with some exceptions. The primary outcome measured was hospital readmission or emergency department/clinic revisit rates within 30 days of treatment initiation. The 30-day readmission/revisit rates were 14.6% and 13.5% for fosfomycin and ertapenem, respectively (i.e., clinical cure rates of 85.4% and 86.5%, respectively), with no difference in outcome for the various fosfomycin dosing regimens. The authors concluded that fosfomycin was noninferior to ertapenem in the treatment of ESBL-associated LUTIs and recommended its consideration as step-down therapy in treating these infections.

In 2016, Jacobson et al. published a retrospective review evaluating the treatment of 71 patients (21 males, 50 females; median age 75 years) treated with oral fosfomycin for UTIs in hospital [11]. Significant comorbidities and/or urologic complications were present in many patients including 52/71 (73.2%) patients with diabetes, 16/71 (22.5%) patients with renal insufficiency, and 8/71 (11.3%) patients with a recent urologic procedure, 38/71 (53.5%) patients were immunocompromised, and 16/71 (22.5%) were receiving chronic systemic steroids. *E. coli* was the most common urinary pathogen isolated. It accounted for 40/71 (56.3%) isolates, 20% (8/40) of which were ESBL-producing *E. coli*. Fosfomycin dosing regimens included 35/71 (49.3%) patients treated with a single 3-gram dose, 10/71 (14.1%) patients treated with 3 grams every 48 hours for a total of 3 doses, and 26/71 (36.6%) patients treated with 3 grams every 72 hours for a total of 3 doses. Clinical cure, defined as resolution of UTI symptoms, occurred in 83% of patients, with no mention of any differences between the various fosfomycin dosage regimens. Relapse, defined as recurrence of the same organism, occurred in 3% of patients. The adverse event rate to fosfomycin was low (4%). The authors concluded that this data supports existing evidence that fosfomycin is a valid option for the treatment of cLUTIs.

In 2017, Giancola et al. published a retrospective evaluation that included 57 patients (19 males, 38 females; median age 79 years) with complicated and/or MDR UTI treated with ≥1 dose of oral fosfomycin [12]. All patients in the study had signs and symptoms of UTI and a positive urine culture. 77.2% (44/57) of patients had at least one complicating factor: 36/57 (40.4%) patients had an infection due to an MDR pathogen and 23/57 (40.4%) patients had both complicated and MDR infections. The authors defined complicating factors for UTIs to include male gender, presence of a Foley or suprapubic catheter, or an anatomical or function abnormality such as neurogenic bladder or a ureteral stent. The most common complicating factors/comorbidities included recurrent UTI in 28/57 (49.1%) patients, Foley or suprapubic catheter in 27/57 (47.4%) patients, and diabetes mellitus in 20/57 (35.1%) patients. Fosfomycin dosing regimens included 26/57 (45.6%) patients treated with a single 3-gram oral dose, 20/57 (35.1%) patients who received 3 grams daily for a total of 3 doses (some every 48 hours and some every 72 hours), and 11/57 (19.3%) patients who received varying regimens ranging from 3 grams once daily or one weekly for 2 to 5 doses (Table 1). Clinical cure was defined as resolution of signs and symptoms during or at the completion of treatment with fosfomycin. Microbiological cure was defined as a negative urine culture after completion of therapy and/or absence of relapse or reinfection. Clinical and microbiological cure rates were 96.4% and 75.0%, respectively, with no mention of any differences between the various fosfomycin dosage regimens. Relapse and reinfection was determined by a repeat urine culture taken at a 30-day follow-up. Relapse was defined as recurrence of the same organism in culture, while reinfection was the presence of a different organism. Relapse and reinfection occurred in 10.0% and 15.0% of patients, respectively. The authors concluded that oral fosfomycin may be effective for the treatment of patients with complicated or MDR UTIs.

In 2018, Cai et al. reported a multicentre retrospective study evaluating the efficacy of oral fosfomycin in the treatment of 35 patients (29 males, 6 females; median age 65.9 ± 8.3 years) with difficult-to-treat catheter-associated UTIs [13]. Difficult-to-treat UTIs were defined as infections where there was no possibility of fluoroquinolone, aminoglycoside, or cephalosporin use due to resistance, failure, or side effects. All patients were initially treated with 3 grams of oral fosfomycin for 2 days and then 3 grams orally every 48 hours for two weeks. 17.1% (6/35) of patients achieved clinical response after the first dose of fosfomycin, 34.2% (12/35) achieved clinical response after two doses, and 37.1% (13/35) achieved clinical response after ≥3 doses; 11.4% (4/35) of patients failed treatment. At follow-up (median time 8 months following therapy), 30/35 patients (85.7%) did not have persistent infection and one patient had demonstrated relapse or reinfection. The authors concluded that oral fosfomycin may be a possible treatment for patients with catheter-associated UTIs.

## 4. Intravenous (IV) Fosfomycin for the Treatment of Patients with Complicated Upper Urinary Tract Infection (cUUTI) or Acute Pyelonephritis (AP)

To date, we are aware of only one published comparative clinical trial evaluating the efficacy of IV fosfomycin in the treatment of upper urinary tract infections, specifically cUUTI and AP. The ZEUS trial, published by Kaye et al. in 2019, compared the efficacy of IV fosfomycin against piperacillin-tazobactam in the treatment of cUUTI and AP [3]. The results of this trial indicated that IV fosfomycin was noninferior to piperacillin-tazobactam in the treatment of cUUTI and AP. The results of the ZEUS trial are summarized in Tables 2 and 3.

The ZEUS trial was a multicentre, randomized, parallel-group, double-blind Phase 2/3 trial designed to assess the efficacy, safety, tolerability, and pharmacokinetics of IV fosfomycin in the treatment of cUUTI and AP in hospitalized patients [3]. The data for this trial was gathered from 92 global sites across 16 countries. The study included a total of 465 patients with suspected or microbiologically confirmed cUUTI or AP. Patients were randomized 1 : 1 to receive either IV fosfomycin or piperacillin-tazobactam. The two dosing regimens were 6 grams IV fosfomycin infused over 1 hour every 8 hours and 4.5 grams piperacillin-tazobactam (4.0 grams piperacillin/0.5 grams tazobactam) infused over 1 hour every 8 hours. Fosfomycin was dose-adjusted in patients with a creatinine clearance <50 and ≥ 20 mL/minute. Dosage adjustment was not needed in the piperacillin-tazobactam group. Patients were treated for 7 days except for patients with concurrent bacteremia, who were treated for up to 14 days at the investigators discretion. Some patients received antibiotic therapy prior to treatment in the trial; however, randomization was stratified based on the region (United States versus rest of world), baseline diagnosis (cUUTI versus AP), and prior antibiotic therapy to minimize confounding. No patients received oral step-down therapy.

The primary objective of the study was to demonstrate noninferiority in the overall success of IV fosfomycin versus piperacillin-tazobactam in the treatment of cUUTI and AP [3]. Overall success was quantitated by a composite of clinical cure and microbiologic eradication at test-of-cure (TOC, day 19–21) in the microbiologic modified intent-to-treat (m-MITT) population. Clinical cure was defined as complete resolution or significant improvement of signs and symptoms such that no further antimicrobial therapy was warranted. Microbiological eradication was defined as baseline pathogen reduction to <10^4^ CFU/mL on urine culture and if applicable, negative on repeat blood culture. Pathogen typing was conducted using blinded, post hoc, pulsed-field gel electrophoresis (PFGE) in order to confirm pathogen specific microbiological eradication/persistence in patients with urinary pathogens present at baseline and at TOC. Secondary objectives of the study were to compare (i) clinical cure rates in the two treatment groups in modified intent-to-treat (MITT), m-MITT, clinically evaluable (CE), and microbiologically evaluable (ME) populations at TOC and (ii) microbiological eradication rates in m-MITT and ME population at TOC. The MITT population included patients receiving any amount of study drug during the trial. The m-MITT population consisted of patients in the MITT population with the presence of ≥1 Gram-negative pathogen in appropriately collected urine or blood culture prior to treatment with the assigned study drug. Patients were considered clinically evaluable (CE) if they met inclusion/exclusion criteria, received ≥9 doses of study drug, and attended appropriate follow-up visits [3]. Patients were considered microbiologically evaluable (ME) if they met MITT and CE criteria and had suitably collected interpretable urine cultures at follow-up visits. Patients with missing urine cultures at follow-up were classified as intermediates and conservatively deemed as failures in the overall success analysis.

Of the 465 patients enrolled in the study, 233 patients were treated with IV fosfomycin, while 231 were treated with piperacillin-tazobactam [3]. The m-MITT population consisted of 184 patients (65 males, 119 females; mean age 49.9 ± 20.9 years) treated with IV fosfomycin and 178 patients (67 males, 111 females; mean age 51.3 ± 20.7 years) treated with piperacillin-tazobactam. Overall success was measured as a composite of clinical/microbiological success rates in the m-MITT population and was 64.7% (119/184) and 54.5% (97/178) in the fosfomycin and piperacillin-tazobactam groups, respectively (Table 2). In patients with cUUTI specifically, overall success rates were superior in those treated with fosfomycin versus those treated with piperacillin-tazobactam; 61.2% (52/85) and 41.7% (35/84), respectively, treatment difference 19.5% (95% confidence interval 3.5–35.5). No significant difference in overall success rates between treatment groups was found in patients with AP; 67.7% (67/99) in the fosfomycin group and 66.0% (62/94) in the piperacillin-tazobactam group. Based on statistical analysis using FDA-agreed upon noninferiority margins, IV fosfomycin was deemed to be noninferior to piperacillin-tazobactam in the treatment of cUUTI and AP.

*E. coli* was the most commonly isolated pathogen in both the fosfomycin and piperacillin-tazobactam groups, followed by *K. pneumoniae* [3]. *E. coli* was isolated in 133/184 (72.3%) and 133/178 (74.7%) of urine cultures in the fosfomycin and piperacillin-tazobactam groups, respectively. Clinical cure and microbiological cure rates per pathogen are recorded in Table 2. The overall clinical cure rate was similar between treatment groups, 90.8% (167/184) and 91.6% (163/178) in the fosfomycin and piperacillin-tazobactam groups, respectively (Table 2). Microbiological eradication rates were 69.0% (127/184) and 57.3% (102/178) in the fosfomycin and piperacillin-tazobactam groups, respectively. Clinical cure and microbiological eradication rates of fosfomycin and piperacillin-tazobactam in isolates with resistant phenotypes are displayed in Table 3. Fosfomycin demonstrated similar clinical cure rates compared to piperacillin-tazobactam in patients with infections due to resistant phenotypes. Microbiological eradication rates in resistant phenotypes, however, appeared to be numerically superior in those treated with fosfomycin (statistical significance unknown). In patients with microbiological persistence after completion of treatment, a greater proportion of patients treated with fosfomycin (19.5%) versus piperacillin-tazobactam (9.7%) had postbaseline growth of baseline pathogens with ≥4 fold increase in MIC to the study drug. In all cases, these patients had complicated disease.

Adverse events were monitored in the safety population throughout the trial [3]. The safety population included all patients who met inclusion/exclusion criteria and received any amount of the study drug. Patients were monitored for changes in baseline laboratory test values, electrocardiogram, and vital signs after initiation of treatment. Specifically, elevations in alanine aminotransferase (ALT), aspartate aminotransferase (AST), alkaline phosphatase (ALP), and total bilirubin were monitored in laboratory test results. Treatment-emergent adverse events occurred in 42.1% of patients treated with fosfomycin and 32.0% of patients treated with piperacillin-tazobactam (statistical significance unknown). The vast majority of these adverse events were asymptomatic changes in blood parameters (e.g., elevation in liver enzymes and/or hypokalemia) and/or gastrointestinal upset (e.g., nausea, vomiting, and/or diarrhea). None of the elevations in aminotransferase enzymes were symptomatic or treatment limiting; in all cases, aminotransferase levels returned to baseline following the end of treatment. Hypokalemia was noted in 30.6% (17.7% mild, 11.2% moderate, and 1.7% severe) of patients treated with fosfomycin and 12.6% (11.3% mild, 0.9% moderate, and 0.4% severe) of patients treated with piperacillin-tazobactam. No serious cardiac adverse events were observed in either treatment arm; however, patients treated with IV fosfomycin were more likely to experience QTcF prolongation (7.3% fosfomycin, 2.5% piperacillin-tazobactam). Serious adverse events were uncommon in both treatment groups (2.1% fosfomycin, 2.6% piperacillin-tazobactam). Only one drug-related adverse event in each group was considered serious: severe hypokalemia in the fosfomycin group and renal impairment in the piperacillin-tazobactam group. There were no deaths in the study. The authors concluded that IV fosfomycin was a safe and effective therapeutic option in the treatment of cUUTI and AP.

## 5. Recommendations for the Treatment of Patients with Complicated Lower Urinary Tract Infection (cLUTI) Using Oral Fosfomycin

To our knowledge, a total of ten studies have been published investigating the use of oral fosfomycin in the treatment of cLUTI [4–13]. It is important to note that these studies varied in their definitions of “complicated” LUTI. As a result, we have comprehensively defined cLUTI to include commonly accepted definitions used in published reports. The majority of these studies were noncomparative; however, Senol et al. [6] and Veve et al. [10] assessed the efficacy of oral fosfomycin in comparison to carbapenems in the treatment of cLUTI (Table 1). The studies we have summarized in Table 1 included a total of 642 patients, ranging from 27 to 146 patients per study. The mean patient age ranged from 50 to 73 years. The majority of patients included in the studies were female (∼65% females, ∼35% males). MDR pathogens, including ESBL-producing *E. coli* and *Klebsiella* spp. were commonly isolated (>300 MDR isolates). Microbiological cure rates ranged from 52.5 to 78.5%, while the clinical cure rates ranged from 68.9 to 94.3%. Relapse and reinfection rates ranged from 0 to 24.4% and 2.9 to 17.1%, respectively. Fosfomycin dosing regimens varied depending on the presence and types of host-complicating factors/comorbidities in study populations. The most common successful regimen reported was 3 grams of oral fosfomycin administered every 48 or 72 hours for a total of three doses. Multidose fosfomycin regimens demonstrated higher rates of clinical success compared to a single dose of fosfomycin, with no additional risk of adverse effects. Comparative trials assessing the efficacy of oral fosfomycin versus IV carbapenems in the treatment of cLUTI have shown no significant differences in outcomes between the agents. Our consensus is that oral fosfomycin is a valid therapeutic option for cases of cLUTIs and difficult to treat LUTIs. Caution is advised in using fosfomycin in organ transplant recipients, especially kidney transplant recipients, and in patients with *Klebsiella* spp. infections, due to increased likelihood of treatment failure. Based on the available data, we recommend individualizing treatment regimens for patients based on the type of pathogen and types/severity of host-complicating factors/comorbidities present.

An oral regimen of 3 grams of fosfomycin given every 48 or 72 hours for 3 doses for patients who have failed previous treatment with another agent, have a MDR pathogen, or cannot tolerate first-line treatment due intolerance or adverse effects may be a good starting point for treatment of cLUTIs. Microbiological cure and clinical cure should be monitored with drug discontinuation in the case of allergy or serious adverse reaction. Extended courses of fosfomycin can be considered in patients who are poorly responsive/nonresponsive to treatment as extended-treatment durations have been shown to be generally well-tolerated.

## 6. Recommendations for the Treatment of Patients with Complicated Upper Urinary Tract Infection (cUUTI) and Acute Pyelonephritis (AP) Using Intravenous Fosfomycin

To our knowledge, the ZEUS trial is the only comparative randomized-controlled trial undertaken to assess the efficacy of IV fosfomycin in the treatment of upper urinary tract infections, specifically cUUTIs and AP. The results of the study demonstrated that IV fosfomycin was noninferior to piperacillin-tazobactam in the treatment of cUUTI and AP.

Our consensus is that the results of the ZEUS trial support IV fosfomycin (at the tested dose 6 grams every 8 hours) as a safe and effective therapeutic option particularly in the treatment of cUUTI (including in patients with concomitant bacteremia) especially in patients infected with *Enterobacterales* with resistance phenotypes including ESBL-producing, CRE, aminoglycoside-resistant, and MDR. A treatment duration of 7 days demonstrated efficacy, but in patients with concurrent bacteremia, effective (and safe) treatment was administered for up to 14 days. Aminotransferase elevation, hypernatremia, hypokalemia, and gastrointestinal upset are the most common adverse events in those treated with IV fosfomycin; however, these adverse events are generally mild, asymptomatic, and transient. We suggest monitoring liver enzymes, sodium and potassium concentrations when treating patients with IV fosfomycin. Patients with persistent infection should have fosfomycin MICs reassessed after treatment as they may benefit from alternative or concurrent agents. More data exploring IV fosfomycin's role as a single or adjunctive agent in the treatment of upper urinary tract infections is needed.

## 7. Using Intravenous (IV) Fosfomycin for Treating Infectious Diseases in Canada

The ZEUS study clearly demonstrated that IV fosfomycin when used alone is a safe and effective therapeutic option in treating upper urinary tract infections, particularly cUUTIs (including in patients with concomitant bacteremia) caused by antimicrobial-resistant *Enterobacterales* [3]. However, we believe that, in Canada, the majority of IV fosfomycin use will be in combination therapy with other antimicrobials [14]. In fact, extensive case reports, case series, cohort descriptive studies, and clinical trials have described the use of fosfomycin in combination with other antimicrobial agents in central nervous system infections, respiratory infections, complicated urinary tract infections, infectious endocarditis and septicemia, osteomyelitis, and soft tissue infections [14, 15]. In these studies, clinical resolution of infections involving fosfomycin treatment occurred in ∼80% of treated patients. Intravenous fosfomycin used in combination with a variety of antimicrobials (e.g., *β*-lactams, carbapenems, glycopeptides, fluoroquinolones, and colistin) has demonstrated additive and frequently synergistic activity *in vitro* [14, 15].

IV fosfomycin's role in Canadian hospitals would be as therapy for patients with infections that have not responded to first- and potentially second-line antimicrobials or in patients that cannot tolerate (due to adverse effects) first- and second-line antimicrobials [14]. IV fosfomycin would primarily be used in combination with *β*-lactams, carbapenems, fluoroquinolones, aminoglycosides, colistin, and glycopeptides/glycolipopeptides for the treatment of MDR *Enterobacterales*, MDR *P. aeruginosa*, MDR methicillin-resistant *Staphylococcus aureus* (MRSA), MDR methicillin-resistant *Staphylococcus epidermidis* (MRSE), and MDR vancomycin-resistant *Enterococcus* (VRE) infections. Using fosfomycin in combination with another antimicrobial should limit the development of resistance to this agent over time. Because of its proven safety, it may be considered for use in preference to potentially toxic agents such as colistin, tigecycline, and aminoglycosides. It may also potentially be used as a carbapenem-sparing agent. In addition, due to its exceptional tissue distribution, it could be used not only for the most common infections such as bacteremia, urinary tract, skin and soft tissue, and respiratory infections, but also for difficult-to-treat infections such as bone infections, meningitis, and invasive ocular infections [15]. Finally, based upon the results of the ZEUS study, IV fosfomycin alone or potentially in combination would be a preferred option for treating complicated UTIs (including in patients with concomitant bacteremia) due to antimicrobial-resistant *Enterobacterales*.

## 8. Conclusions

We have reviewed the available literature citing the use of oral fosfomycin in the treatment of cLUTIs. Collectively, among 642 patients, microbiological cure rates ranged from 52.5 to 78.5%, while clinical cure rates ranged from 68.9 to 94.3%. Two comparative trials documented oral fosfomycin to be noninferior to IV carbapenems in the treatment of cLUTIs. Collectively, these studies support a regimen of 3 grams of oral fosfomycin administered every 48 or 72 hours for 3 doses for patients who have failed previous treatment, have an MDR pathogen, or cannot tolerate first-line treatment due to intolerance or adverse effects. Caution is advised in using fosfomycin in organ transplant recipients, especially kidney transplant recipients, and in patients with *Klebsiella* spp. infections, due to increased likelihood of treatment failure. We have also reviewed results from a recent clinical trial, known as the ZEUS study, which comparatively assessed the efficacy and safety of IV fosfomycin versus piperacillin-tazobactam in the treatment of cUUTI and AP (including in patients with concomitant bacteremia). The results of this trial demonstrated that IV fosfomycin was noninferior to piperacillin-tazobactam in treating these infections and may, in fact, be superior when treating cUUTIs especially when caused by antimicrobial-resistant pathogens. Based on the ZEUS study, our consensus is that at the tested dose of 6 grams every 8 hours for 7 days (14 days in patients with concurrent bacteremia), IV fosfomycin is a safe and effective therapeutic option for the treatment of upper urinary tract infections and may be of particular use in the treatment of patients with cUUTI (including in patients with concomitant bacteremia) caused by an antimicrobial-resistant pathogen. Further research is required to fully elucidate the roles of oral and IV fosfomycin when used as a single agent or in combination with other antimicrobials agents in treating cLUTIs, cUUTIs, and AP.

## Figures and Tables

**Table 1:** Oral fosfomycin for the treatment of patients with complicated lower urinary tract infection (cLUTI).

Study	Number of patients	Mean age (years)	Gender (male/female)	Complicating factors/comorbidities	Pathogens	Dosing regimen	Microbiologic cure (%)	Clinical cure (%)	Relapse rate (%)	Reinfection (%)
Moroni et al. [4]	49	—	20/29	—	—	21 patients—3 grams single dose; 28 patients—3 grams once daily for 2-3 days or once daily for 11 days (few cases) multi-dose	—	Single dose, 57.1% Multidose, 82.1%	—	—

Pullukcu et al. [5]	52	55	25/27	∼1.7	All ESBL-producing *E. coli*	3 grams every other day for 3 doses	78.5%	94.3%	0%	10.7%

Senol et al. [6]	27	58	13/14	∼1.7	All ESBL-producing *E. coli*	3 grams every other day for 3 doses	59.3%	77.8%	6.3%	6.3%

Neuner et al. [7]	41	62	19/22	∼4.4	All MDR urinary pathogens	3 grams for 2.9 ± 1.8 doses	58.5%	—	24.4%	17.1%

Qiao et al. [8]	146	50	—	—	—	3 grams every other day for 3 doses	74.6%	70.5%	14.6% relapse or reinfection	

Matthews et al. [9]	75	73	18/57	∼1.2	52/75 *E. coli*; 31/52 ESBL-producing *E. coli*	53 patients, 3 gram single dose; 8 patients, 3 grams for two doses 14 patients, 3 grams for ≥3 doses	52.5%	68.9% “functional cure”	—	10.7%

Veve et al. [10]	89	69	23/66	∼3.7	All ESBL-producing *E. coli*	12 patients, 3 grams single dose; 20 patients, 3 grams every 48 hours for ∼3 doses; 55 patients, 3 grams every 72 hours for ∼3 doses 2 patients, 3 grams daily for ∼4.5 doses	—	85.4%	—	—

Jacobson et al. [11]	71	75	21/50	—	40/71 *E. coli*; 8/40 ESBL-producing *E. coli*	35 patients, 3 grams single dose; 10 patients, 3 grams every 48 hours for 3 doses 26 patients, 3 grams every 72 hours for 3 doses	—	83.0%	3.0%	—

Giancola et al. [12]	57	79	19/38	∼2.2	36/57 MDR pathogens	26 patients, 3 grams single dose; 20 patients, 3 grams every 48–72 hours for 3 doses 11 patients, varying regimens	75.0%	96.4%	10%	15%

Cai et al. [13]	35	66	28/6	>1	23/35 *E. coli*; 17/35 ESBL-producing	3 grams once daily for 2 days followed by 3 grams every 48 hours for 2 weeks	—	85.7%	2.9% relapse or reinfection		

Table created from data in references [4–13]. Complicating factors/comorbidities, average number of complicating factors/comorbidities per patient (see definition of complicated UTI). ESBL: extended-spectrum *β*-lactamase; MDR: multidrug-resistant (resistant to at least one agent in ≥3 antimicrobial classes). Microbiological cure defined as eradication of urinary pathogen as documented by urine culture at completion of therapy (^*∗*^exception: references [7, 12] define microbiological cure, as a negative urine culture after completion of therapy and/or absence of relapse or reinfection). Clinical cure defined as resolution of UTI symptoms including dysuria, urgency, and frequency. “Functional cure” was defined by Matthews et al. as (i) evidence of microbiological cure and/or (ii) no follow-up sample available presumably due to clinical cure. Relapse defined as reappearance of same causative pathogen in urine culture at follow-up. Reinfection defined as presence of a different urinary pathogen in urine culture than at initial presentation at the time of follow up.

**Table 2:** Randomized comparative trial data of ZTI-01 (IV fosfomycin) versus piperacillin-tazobactam in the treatment of patients with complicated urinary tract infection (cUTI) and acute pyelonephritis (AP).

Trial description	Study type	Total subjects	Treatment regimens	Primary outcome measure	Secondary outcome measure	
Author: Kaye et al. [3] *Clinical Trial Registration.* NCT02753946	Phase 2/3 randomized trial	465	Experimental: Fosfomycin 6 grams IV (*t*' 1 h) every 8 hours for 7–14 days	(i) Overall response^a^ was defined as a composite of clinical cure and microbiologic eradication at TOC in the m-MITT population (*N*)	(i) Clinical cure^b^ rates in the 2 treatment groups in MITT, m-MITT, CE, and ME populations (*N*) at TOC (ii) Microbiological eradication^c^ rates in m-MITT and ME population at TOC	
			Comparator: Piperacillin-tazobactam 4 grams piperacillin/0.5 grams tazobactam IV (infused over 1 hour) every 8 hours for 7–14 days	Results *n*/*N* (%) Fosfomycin: cUTI: 52/85 (61.2%) AP: 67/99 (67.7%) Total: 119/184 (64.7%) Piperacillin-tazobactam: cUTI: 35/84 (41.7%) AP: 62/94 (66.0%) Total: 97/178 (54.5%)	Results *n*/*N* (%) Clinical cure overall	Fosfomycin: 167/184 (90.8%) Piperacillin-tazobactam: 163/178 (91.6%)
					Clinical cure per pathogen *E. coli* *K. pneumoniae* *Proteus mirabilis* *Enterobacter cloacae* species complex *P. aeruginosa* Microbiological eradication Microbiological eradication per pathogen *E. coli* *K. pneumoniae* *P. mirabilis* *E. cloacae* species complex *P. aeruginosa*	Fosfomycin: 120/133 (90.2%) Piperacillin-tazobactam: 120/133 (90.2%) Fosfomycin: 25/27 (92.6%) Piperacillin-tazobactam: 25/25 (100%) Fosfomycin: 8/9 (88.9%) Piperacillin-tazobactam: 3/5 (60.0%) Fosfomycin: 8/9 (88.9%) Piperacillin-tazobactam: 3/3 (100%) Fosfomycin: 8/8 (100%) Piperacillin-tazobactam: 9/9 (100%) Fosfomycin: 121/184 (65.8%) Piperacillin-tazobactam: 100/178 (56.2%) Fosfomycin: 87/133 (72.9%) Piperacillin-tazobactam: 84/133 (63.2%) Fosfomycin:18/27 (66.7%) Piperacillin-tazobactam: 14/25 (56.0%) Fosfomycin: 8/9 (88.9%) Piperacillin-tazobactam: 1/5 (20.0%) Fosfomycin: 6/9 (66.7%) Piperacillin-tazobactam: 3/3 (100%) Fosfomycin: 3/8 (37.5%) Piperacillin-tazobactam: 4/9 (44.4%)

Table adapted from reference [3]. TOC, test of cure (day 19–21); m-MITT, microbiologic modified intent to treat population; MITT, microbiological intent to treat; CE, clinically evaluable; ME, microbiologically evaluable; *N,* total number of patients in specified cohort; *n*, number of patients achieving specified outcome. ^a^Overall response measure defined as complete resolution or significant improvement of signs and symptoms such that no further antimicrobial therapy is warranted; baseline pathogen reduction to <10^4^ CFU/mL on urine culture, if applicable on repeat blood culture. ^b^Clinical cure defined as complete resolution or significant improvement of signs and symptoms such that no further antimicrobial therapy is warranted. ^c^Microbiological eradication defined as a urine culture or blood culture, if applicable, demonstrating that the bacterial pathogen identified at baseline has decreased from ≥10^5^ CFU/mL to <10^4^ CFU/mL.

**Table 3:** Clinical cure and microbiologic eradication outcomes of ZTI-01 (intravenous fosfomycin) versus piperacillin-tazobactam in patients with complicated urinary tract infections (cUTI) and acute pyelonephritis (AP), due to baseline pathogens with resistant phenotypes.

Phenotype	Clinical cure^a^		Microbiologic eradication^b^	
	Fosfomycin *n*/*N* (%)	Piperacillin-tazobactam *n*/*N* (%)	Fosfomycin *n*/*N* (%)	Piperacillin-tazobactam *n*/*N* (%)
ESBL	52/56 (93%)	51/55 (93%)	32/58 (55%)	27/57 (47%)
Aminoglycoside-resistant	29/30 (97%)	29/31 (94%)	20/30 (67%)	12/32 (38%)
CRE	9/9 (100%)	11/13 (85%)	5/9 (56%)	4/13 (31%)
MDR	34/37 (92%)	28/31 (90%)	20/37 (54%)	12/33 (36%)

Table adapted from reference [3]. ESBL, extended-spectrum *β*-lactamase (≥2 *μ*g/mL MIC for aztreonam, ceftazidime, or ceftriaxone); aminoglycoside-resistant (gentamicin ≥8 *μ*g/mL MIC or amikacin ≥32 *μ*g/mL MIC); CRE, carbapenem-resistant *Enterobacterales* (≥4 *μ*g/mL MIC for imipenem or meropenem); MDR, multidrug-resistant (resistant to at least one agent in ≥3 antimicrobial classes); *N,* total number of patients in specified cohort; *n*, number of patients achieving specified outcome. ^a^Clinical cure defined as complete resolution or significant improvement of signs and symptoms such that no further antimicrobial therapy is warranted. ^b^Microbiological eradication is defined as a urine culture or blood culture, if applicable, demonstrating that the bacterial pathogen identified at baseline has decreased from ≥10^5^ CFU/mL to <10^4^ CFU/mL.
